# Adolescents’ self-efficacy and digital health literacy: a cross-sectional mixed methods study

**DOI:** 10.1186/s12889-022-13599-7

**Published:** 2022-06-20

**Authors:** Melody Taba, Tiffany B. Allen, Patrina H.Y. Caldwell, S. Rachel Skinner, Melissa Kang, Kirsten McCaffery, Karen M. Scott

**Affiliations:** 1 Specialty of Child and Adolescent Health, Faculty of Medicine and Health, The University of Sydney, Sydney, NSW Australia; 2 Sydney Health Literacy Lab, School of Public Health, Faculty of Medicine and Health, The University of Sydney, Sydney, NSW Australia; 3 The Children’s Hospital at Westmead, Westmead, NSW Australia; 4 Specialty of General Practice, Faculty of Medicine and Health, The University of Sydney, Sydney, NSW Australia

**Keywords:** Adolescents, Digital health literacy, eHealth literacy, Critical health literacy, Online health information, Digital health information, Internet, Information seeking behaviour, Websites, Social media

## Abstract

**Background:**

The internet and social media are increasingly popular sources of health information for adolescents. Using online health information requires digital health literacy, consisting of literacy, analytical skills and personal capabilities such as self-efficacy. Appraising trustworthiness and relevance of online health information requires critical health literacy to discriminate between sources, critically analyse meaning and relevance, and use information for personal health. Adolescents with poor digital health literacy risk using misinformation, with potential negative health outcomes. We aimed to understand adolescents’ contemporary digital health literacy and compared self-efficacy with capability.

**Methods:**

Adolescents (12–17 years) completed an eHEALS self-report digital health literacy measure, a practical search task using a think-aloud protocol and an interview to capture perceived and actual digital health literacy. eHEALS scores were generated using descriptive statistics, search tasks were analysed using an observation checklist and interviews were thematically analysed based on Social Cognitive Theory, focussing on self-efficacy.

**Results:**

Twenty-one participants generally had high self-efficacy using online health information but perceived their digital health literacy to be higher than demonstrated. They accessed online health information unintentionally on social media and intentionally via search engines. They appraised information medium, source and content using general internet searching heuristics taught at school. Information on social media was considered less trustworthy than websites, but participants used similar appraisal strategies for both; some search/appraisal heuristics were insufficiently nuanced for digital health information, sometimes resulting in misplaced trust or diminished self-efficacy. Participants felt anxious or relieved after finding online health information, depending on content, understanding and satisfaction. They did not act on information without parental and/or health professional advice. They rarely discussed findings with health professionals but would welcome discussions and learning how to find and appraise online health information.

**Conclusions:**

Whilst adolescents possess many important digital health literacy skills and generally feel self-efficacious in using them, their critical health literacy needs improving. Adolescents desire increased digital health literacy so they can confidently appraise health information they find online and on social media. Co-designed educational interventions with adolescents and health providers are required.

**Supplementary Information:**

The online version contains supplementary material available at 10.1186/s12889-022-13599-7.

## Introduction

Adolescence is a key developmental phase involving multiple physical, cognitive and psychosocial changes and increasing independence, as adolescents learn about themselves and their world and establish health behaviours that they carry into adulthood [1, 2]. Adolescents are navigating this unique period in an era when digital media is ubiquitous in their everyday lives, including for entertainment, learning and information access [3]. In 2020, Australian adolescents aged 12–17 years spent an average of 14.4 hours a week online and used an average of four different social media platforms [4]. Increasingly, adolescents are turning to online sources like websites and social media for health-related information on topics such as sexual health, mental health, chronic and infectious disease, fitness and nutrition [5, 6]. A survey of Australian adolescents found 78% and 77% of participants reported using websites and social media respectively when seeking health-related information [7]. Adolescents favour the ease of access, convenience and privacy of the internet in comparison to traditional sources of health information [8, 9]. This level of accessibility and abundance of information can empower adolescents as active agents in their health and address feelings of information poverty [10].

However, the unregulated internet can host biased, inaccurate and poor-quality health information, which can lead to negative health outcomes in adolescents if acted upon [7, 11–13]. Websites and social media can rapidly spread health misinformation, as demonstrated by the COVID-19 ‘infodemic’ [14]. Other risks and harms of online health for adolescents can include becoming overly obsessed with their bodies’ shape and size when using self-tracking technologies and comparing their bodies with social media influencers they follow [15]. Thus, using online health information safely and effectively requires a level of digital health literacy, with which adolescents, despite being digital natives, may not be equipped [16, 17].

### Digital health literacy

Grounded in the digital context, this study builds on Nutbeam’s [18] broad definition of health literacy: the ability to access, appraise, understand and apply health information to address a health problem. Nutbeam identifies three progressive levels of cognitive and social skills required by individuals to apply health information to achieve health outcomes in different circumstances. ‘Functional health literacy’ describes the basic reading and writing skills and knowledge of health conditions and systems, such as the internet, needed to obtain and apply health information to a limited range of circumstances. ‘Interactive health literacy’ describes more advanced literacy and personal skills required to extract information and meaning from different forms of communication and apply new information to changing circumstances. ‘Critical health literacy’ describes the most advanced cognitive and social skills that enable individuals to discriminate between varying sources of information, critically analyse meaning and relevance, and use information to exert greater control over personal health.

Previous studies have indicated adolescents can face functional challenges when using online health information, including understanding medical terms and constructing search terms, interactive challenges with applying information to personal health concerns, and critical challenges, including discerning relevance and trustworthiness of information [19–21]. Adolescents with weak digital health literacy may be at risk of finding inaccurate information and being influenced by groups with self-serving interests [11]. This can proliferate incorrect or biased beliefs and behaviour, leading to anxiety and incorrect self-diagnosis and treatment, potentially resulting in poor or even fatal health outcomes [11, 22–24]. Social media poses new digital health literacy challenges for adolescents. Young people may have decreased perceptions of potential risks of health information on social media because they expect to find entertaining content on a platform they use predominately to communicate with friends [25].

Given the permeation of the internet and social media in adolescents’ daily lives, it has become increasingly important to understand adolescents’ digital health literacy and ensure they are equipped with the required skills to navigate online health information. The evidence base is in need of strengthening due to the ever-changing digital health information landscape, more recently affected by the COVID-19 pandemic.

### Social cognitive theory and self efficacy

We adopt Social Cognitive Theory (SCT) [26] as the theoretical underpinning for our understanding of adolescent digital health literacy. SCT holds that learning, functioning and actions result from a dynamic and reciprocal triadic interaction amongst personal, environmental and behavioural factors. Viewed through the lens of SCT, adolescents’ digital health literacy can be understood by the interplay between personal understandings of and attitudes towards online health information, the online health information environment and behaviours involving online health information.

SCT holds the concept of self-efficacy as a central tenet. An individual’s trust in and perception of their capabilities is required for successful outcomes [27]. Self-efficacy explains that health behaviours are sustained when a person believes they are capable of executing a desired behaviour, for example via performance attainment when past success informs current behaviour [28]. Self-efficacy is useful for understanding adolescents’ digital health literacy capabilities [29, 30]. It explains adolescents’ information-seeking motivation, behaviour and awareness of appraisal and application skills. In this study, our aim was to apply SCT, and self-efficacy specifically, as a lens through which to identify the contemporary digital health literacy capacity of adolescents (aged 12–17 years) by asking the following research questions:

RQ1. How do adolescents perceive their ability to access, appraise, understand and apply online health information?

RQ2. How does this perception compare with their demonstrated digital health literacy when using online health information?

## Methods

This cross-sectional study utilised a mixed-method design, involving quantitative measurement of digital health literacy and a real-time observed health information search task followed by a qualitative semi-structured interview. Through mixed methods, we aimed to triangulate the data to enhance credibility and ensure comprehensive and insightful understanding of the findings [31].

### Sample

We recruited participants living in Australia aged 12–17 years (i.e., secondary school age). Purposive sampling was used to obtain a diverse cross-section of participants in age, gender, presence/absence of health condition and experience with online health information. Participants were recruited through multiple avenues: (1) advertising via the Sydney Children’s Hospitals Network’s communication channels (social media, newsletters, physical noticeboards); (2) at The Children’s Hospital at Westmead by approaching in-patients identified by nurse unit managers based on English-speaking ability and emotional well-being (i.e., adolescents who had just heard bad news were not approached); (3) through Australian youth community organisations (Girl Guides Australia, Multicultural Youth Advocacy Network and Youth Action). Participants were recruited until data saturation was reached (i.e., the point at which no new themes were observed) [32]. A parent or guardian provided informed consent and participants provided informed assent to participate in the study.

### Data collection

All data were collected online, given physical distancing restrictions due to COVID-19 in 2020. Data were collected by MT and TA, public health researchers with experience collecting and generating data with young people. Study methodology, search task and the interview guide were piloted with two non-participant adolescents prior to data collection.

Participants completed an online demographic survey for age, gender, postcode, frequency of internet use, mother tongue and presence of chronic health condition. They subsequently completed an online eHEALS measure, a validated digital health literacy instrument measuring self-reported knowledge, comfort and skills in finding, evaluating and applying online health information to health problems [33]. The instrument consisted of eight statements about online health (with two supplementary items), rated on a five-point Likert scale with responses ranging from strongly disagree (1) to strongly agree (5).

Participants completed a health information search task using private video link on their personal devices. They shared their screens and searched for online health information using a provided health scenario, based on previous research [34]. The scenario consisted of common symptoms (“tummy” pains, unintentional weight loss and bloating) that could underlie numerous diseases, mimicking a real-life situation. Participants were instructed to complete the search as if they were experiencing these symptoms whilst verbalising their search and appraisal decisions, following a concurrent ‘think-aloud’ protocol [35].

Semi-structured interviews, informed by SCT and previous research [34], followed immediately after the search task, using the same private video link. Participants were asked about their understandings, beliefs and attitudes towards online health information (web-based and on social media), search and appraisal abilities and use of online health information (Additional file 1). Participants received a A$30 gift card upon completion as compensation for their time and expertise.

Each search and interview took on average 40.2 min (SD = 18.2 min) to complete. The task screenshare was video- and audio-recorded; interviews were audio-recorded then transcribed verbatim.

### Data analysis

eHEALS responses were quantitatively analysed to calculate an average score for each participant and an average score for the cohort. We followed other studies with similar target populations [30] and considered eHEALS scores above 3.5 out of 5 to represent high digital health literacy. Video recordings were analysed using an observational checklist based on previous research [34] (Additional file 2). Transcripts and observational checklists were thematically analysed using Framework analysis [36]. MT, TA and KS completed independent data ‘familiarisation’ (i.e., immersion in all data recordings, transcripts and field notes) concurrently with data collection. Independent findings were discussed and a consensus was reached before developing and refining a thematic framework informed by SCT. Transcripts were then ‘indexed’ (coded) line-by-line using Dedoose software (Version 9.0.17, SocioCultural Research Consultants, Manhattan Beach, CA, USA). The framework was discussed and modified throughout to ensure themes were grounded in the data. Five transcripts (20%) were ‘double-coded’ by MT and TA and read by KS to ensure similar judgements regarding the data analysis, with conflicting findings discussed until a consensus was reached. A thematic schema was subsequently devised with key themes and subthemes from the thematic framework, taking into consideration the research questions. The analysis is presented below, with illustrative quotations of key points, in which participants are identified by a number; self-described gender and age included.

## Results

The research was conducted with 21 participants: sixteen females and five males; fifteen aged 12–14 years and six aged 15–17 years (mean age = 14.2 years, SD = 1.6 years) (Table 1). Eight used the internet constantly during the day, eleven used it several times a day, with the remaining using it once a day or several times a week.

**Table 1:** Participant demographics (*n* = 21)

Trait	Value	n	%
eHEALS mean score	3.68 (SD 0.47)		
Age (years) mean	14.24 (SD 1.57)		
Age (years)	12	2	10
	13	6	29
	14	7	33
	15	0	0
	16	3	14
	17	3	14
Gender	Female	16	76
	Male	5	24
Recruitment site	Youth organisations	13	62
	Hospital	8	38
Residence	Metropolitan	16	76
	Regional	5	24
Daily internet use	Constantly	8	38
	A few times	11	52
	Once	1	5
	Weekly	1	5
First language	English	18	86
	Other	3	14
Chronic Illness	Yes	4	19
	No	17	81

Overall, participants perceived themselves to have relatively high digital health literacy, rating themselves at a mean eHEALS score of 3.7 (SD = 0.5) out of 5, indicating a strong sense of self-efficacy. The eHEALS supplementary items indicated the majority (62%) believed the internet is useful in health decisions, and 71% believed it was important for them to access health information on the internet.

The total time spent searching in the practical search task ranged from 3 to 18 min (mean = 7.9 min, SD = 3.8 min) and total number of webpages visited as part of the search task ranged from 1 to 8 pages (mean = 3.3, SD = 1.7). From the provided scenario, participants searched for tummy pains 76% of the time, bloating 76% of the time and weight loss 71% of the time.

We identified four main themes from the interviews and search task: accessing, appraising, understanding and applying online health information.

### Accessing online health information

Participants accessed online health information by two methods; unintentionally finding it on social media or intentionally searching for it using a search engine.

#### Unintentionally finding online health information

Participants reported encountering health information on social media simply by virtue of being users of platforms like Instagram, Snapchat and TikTok. Even if they were not intentionally searching for health information, it appeared on their feeds because they followed other users who interacted with or created the content. Generally, this type of information was posted by social media influencers, but some participants reported their offline friends posting about health on social media.

A friend posted an Instagram story: ‘Click this link to see if you have depression’. I did the linked survey, and it turns out I don’t! I mean, I wasn’t wondering if I had depression, but at the same time it was good to know I don’t. (P11 - Male, aged 14)

Participants reported seeing diet, exercise and mental health information on social media feeds. They also reported seeing a large amount of COVID-19-related content, ranging from general facts to health promotion advice, plus “conspiracy” or “anti-COVID” advice. COVID-19 content was often shared by government agencies, third party advertisers, celebrities and influencers.

Every day people post about COVID-19. There’s people that post about how it’s affecting countries and why it’s bad. And some people posting about what we can do to fix it and daily reminders for us to wear masks. (P20 - Female, aged 13)

Accessing health information on social media unintentionally was considered useful as participants learn about health opportunistically:

It’s useful because I’m on social media a lot. Instead of just looking up random health facts, which you wouldn’t necessarily think to do, it just comes up on your feed so it’s more accessible for me. (P10 - Female, aged 14)

#### Intentionally searching for online health information

Participants also intentionally accessed online health information using search engine Google, both in the search task and their personal lives. Searching frequency varied: some searched occasionally, others weekly. Generally, they searched when they experienced symptoms, following onset of symptoms or when these persisted.

I search up whenever I feel crap. (P7 - Male, aged 16)

Depending on the severity of symptoms, which participants assessed based on pain levels and search results, they would alert their parents and ask for help. In these cases, they believed they could not address their concerns alone and felt their capabilities could be improved with assistance from a trusted adult. However, if the participant deemed the symptoms to be severe, they bypassed the search and immediately asked for help from their parents, who would ascertain if they required professional help.

Usually if I search something and it was a bit alarming, I would go to a parent and say, “I think this is happening. I’m not sure, though. Can you just help me research it just in case?” (P5 - Female, aged 14)

Participants explained their searching behaviour was also motivated by larger health events, namely information-seeking about COVID-19. Participants may also search before a health appointment to familiarise themselves about a health issue and “feel prepared for the appointment”, indicating an interest in improving their self-efficacy and actively participating in their healthcare.

#### Search strategies

Participants reported they had developed routine search strategies for finding online health information and demonstrated these during the search task. Regardless of their eHEALS score, participants conducted the search task in a similar way.

As seen in Table 2, participants either aimed to identify a specific condition indicated by the search task symptoms, its cause or treatment, or simply find more information. In follow-up interviews, participants explained they were often motivated to search for validation or reassurance in case they “were stressing over a simple random headache.”

 In the task, participants searched by phrasing a question around the symptoms (e.g. “What does it mean when…”) or using the symptoms as key words for the search. They reported rarely self-diagnosing before searching but considered their personal context when entering search terms. For example, one participant mentioned she would have entered task search terms differently had she been menstruating.

During the task, participants scanned the search results page for key words that matched the provided symptoms and included “health” or “medical”. The majority selected government and official health websites (e.g. Mayoclinic and Healthline) and the rest selected other health-related websites like news articles (e.g. Medical News Today). Most chose the first piece of information available in the results page that was not an advertisement. In some cases, this was a Google Featured Snippet or Suggestion, rather than a link to an external website. Participants confirmed this was usual practice, with many believing that websites listed further down the results list were less relevant and “less professional”:

Google puts the best websites at the top of the page. (P10 - Female, aged 14)

Participants stopped searching when they felt satisfied with the search results, demonstrating a sense of self-efficacy. For most, this occurred when they had cross-checked information on a few websites (most completed additional searches to cross-check and look-up more information). Participants explained, “there wasn’t much point in going further” beyond a few websites; they were disinterested in exhausting all possible information. Nevertheless, some were not satisfied until they had found a remedy or diagnosis that matched the symptoms in the task.

Many participants reported developing their searching strategies from “internet searching basics” taught in school for academic assignments. Some had parents assist them but rarely searched for online health information with friends or learnt about searching from friends.

Participants observed that their search in the task may be different to “real life searches”, given the hypothetical scenario. Their search time may be longer if they were particularly anxious or in pain, and they sometimes “search differently” depending on the symptoms. For example, a 13-year-old female participant explained they use the image search function for a dermatological issue, whilst a 17-year-old female participant explained that for a mental health concern, they would directly navigate to the Australian youth mental health organisation website headspace. This self-awareness of search strategy indicates a sense of self-efficacy.

### Appraising trustworthiness of online health information

#### Appraisal of medium

The online medium of the health information (web-based or social media) was considered important in appraisal of trustworthiness. Health information on social media was considered potentially less trustworthy than websites as “anyone can write anything”. Many raised concerns about the potential for misinformation to be spread by users without a health background. Despite this, participants reported assessing health information on social media in a similar way to information on websites. A strategy unique to social media, however, was the consideration of “Likes” on a social media post in ascertaining trustworthiness:

If something on social media works, then I’ll definitely give it a ‘Like’. When you see a lot of ‘Likes’ and comments saying that, ‘Oh, this thing helps’, then you know it’s reliable and trustworthy because a lot of people experienced it and tried it too. (P9 - Female, aged 17)

#### Appraisal of source

Participants considered that health information on government websites, specifically Australian, was always trustworthy. Participants believed that sponsored and advertising content on social media or web search results was almost always untrustworthy. Some also said news articles and blogs were untrustworthy as a source of online health information.

When using search engine results, many assessed the domain in the URL, stating those with .org and .gov were more trustworthy than those with .com. Participants explained this strategy was taught in schools as a “blanket rule” for internet searching. However, whilst many acknowledged the role of the domain in assessing trustworthiness, many did not use this strategy during the search task. In follow-up interviews, participants reported they did not always note the URL, only checking it if website content indicated it was from an untrustworthy source:

I don’t always look at the domain of the website, to be honest, unless it’s a really dodgy site. You know when it’s .gov or .org, it’s reliable…we were taught that at school. (P7 - Male, aged 16)

Country of origin was also considered important in URL appraisal, especially regarding information about COVID-19. Many believed Australian websites were more trustworthy than American websites (including American government websites) due to perceptions that the USA’s pandemic response was poor.

Familiarity with information source was also important for these adolescents. Participants often navigated to websites they had previously used or heard of as they believed they would have trustworthy or relevant information. Commonly used websites included government health websites such as New South Wales Health, Healthline and Mayoclinic.

#### Appraisal of content

Before clicking on links in search results, participants gleaned the content through the language in titles and key words in website previews. This was to decipher whether the website contained trustworthy and/or relevant information. Participants reported avoiding links with “click-bait” titles; in the search task these were dismissed as untrustworthy and unlikely to contain professional health advice.

Usually I don’t really trust the attention-grabbing titles like ‘Eight warning signs that you’re losing weight too quickly’, because it seems more like a news article trying to get my attention rather than something that’s actually reliable. (P4 - Male, aged 17)

Participants assessed the content of health information by focusing on layout and language cues, scanning health information for relevant keywords. They reported high-quality websites used plain language with keywords, summaries and bullet points. Websites with obvious advertisement banners raised questions about trustworthiness.

The ad at the top of the page looks a bit like a scam to me, like ads for ‘Wish’ [e-commerce platform]. Reliable sources usually don’t have to advertise. (P2 - Female, aged 17)

Participants considered authors of information in their content appraisal, whether on a website or social media. Many identified that authors have biases or agendas that may be present in health information. Websites authored by well-known organisations or medical professionals were considered trustworthy compared with those authored or shared on social media by unidentified and/or unqualified individuals. During the search task, participants pointed out credentials (e.g., MD, PhD) beside author names as indicators of trustworthiness. However, one 13-year-old male participant explained that he did not know "what the letters next to the name mean”, indicating use of strategies without knowledge of how they would lead to trustworthy information.

**Table 2:** Adolescents’ search and appraisal strategies

	Search/appraisal stage	Strategies	Percentage and number (n) who demonstrated strategy in search task (*n* = 21)
Starting search	Search engine selection	Using Google or default search engine	100 (21)
	Aim	To identify specific condition/cause	57.1 (12)
		To identify treatment	28.6 (6)
		To identify general information	14.3 (3)
	Motivation	To gather information	
		For validation/reassurance	
	Search terms	Phrasing symptoms into question	57.1 (12)
		Using symptoms for a key word search	23.8 (5)
		Boolean terms	4.8 (1)
Selection of information	Selecting relevant result	Scanning for key words in search result page	
		Scanning for “health” or “medical” in search result page	
	Selecting trustworthy result	Selecting website over social media	95.2 (20)
		Selecting Google Featured Snippet	52.4 (11)
		Selecting top search result (excluding Featured Snippets)	33.3 (7)
		Avoiding ads or sponsored websites	100 (21)
		Selecting .gov rather than .com	23.8 (5)
		Selecting other official websites	47.6 (10)
		Selecting familiar websites	23.8 (5)
Appraisal of information	Appraising trustworthiness	Considering layout and design	33.3 (7)
		Considering author	28.6 (6)
		Cross-checking	95.2 (20)
	Understanding	Searching difficult or confusing terms	23.8 (5)
	Appraising relevance	Dismissing information about severe disease	28.6 (6)
Ending search	Finishing search	After cross-checking	95.2 (20)
		When satisfied with findings	100 (21)

### Understanding online health information

Participants reported varying levels of understanding of the health information they found online. Very few reported always understanding the information; many had trouble understanding “a couple words or sentences” or large amounts of information. The most common difficulty was due to medical and scientific terminology. Many reported investigating “confusing” or “hard” terms in a subsequent search or seeking assistance from parents; others searched websites with simpler terminology. Some reported stopping searching entirely when they were overwhelmed by complex terms.

A lot of the time there will be a couple words that I won’t understand and I look them up. But if I find a source with good information, but I don’t understand it, I will usually refrain from using that information just because I might understand it in the wrong context. I wouldn’t just use the parts that I do understand in case it’s not exactly what I thought it was. (P12 - Female, aged 14)

Participants understood health information with clear, simple explanations. However, if the language was “too simple”, they may question its trustworthiness. Health information on social media was reported to be easier to understand than health-focussed websites and therefore preferable for some.

Information is presented in a very direct way on social media. If you look up on websites, it’s very long articles with big words that I, as a 17-year-old, wouldn’t understand as much…which is why I usually just go on Facebook, because it’s easier to read and understand. (P9 - Female, aged 17)

#### Emotional impact

Participants felt dissatisfied when they could not understand or find health information. One 14-year-old female explained it was “frustrating when you don’t figure it out” after spending time searching, whilst another 14-year-old female would feel “hopeless” after not attaining the information they wanted. Others felt anxious and stressed when they did not find a satisfying answer to their problem or could not understand the information. If performance attainment was not reached following extensive time or effort, these feelings were exacerbated. Another 14-year-old female felt frustrated if they clicked on more than two websites and could not find what they were seeking.

Conversely, participants felt satisfied once they found useful and understandable health information. They felt relieved when they found information that signalled “it’s going to be OK”. They also felt reassured finding others who had experienced similar symptoms via user-generated sources, such as blogs, YouTube videos and Instagram accounts:

Because YouTube and blogs are based on people’s experience of problems, I can find someone who has faced my problem. It can be very helpful to hear about their experience and what has helped them. (P9 - Female, aged 17)

Participants’ emotional state was dependent on their perceived level of performance attainment: when they found information they deemed accurate and helpful, they felt satisfied and pleased. This sense of attainment and self-efficacy was enhanced if the trustworthiness and relevance of the information was confirmed by parents and/or health professionals.

Humans are built to build trust, so after having a positive outcome from online researching, it led me to understand that although some information is a little bit untrustworthy online, a lot of it can also help you. (P12 - Female, aged 14)

#### Beliefs about value

Participants indicated positive and negative aspects of searching for health information online. A key benefit was accessibility as they could find health information quickly and easily without needing to consult with parents or health professionals. This enabled them to improve their self-efficacy and as one 14-year-old female said, “Catch a condition in an earlier stage so it doesn’t progress and get worse”.

Online health information was considered ideal when dealing with sensitive health conditions for adolescents “who don’t feel comfortable asking their parents about health stuff”. Searching online allowed for privacy to learn about health concerns, such as sexual health, without having to speak with an adult. Online health information was often the “first port of call” when experiencing symptoms. Some explained that good online health information could replace a health professional for minor ailments, especially when considering the cost, time and effort involved. Online health information “is already there” and “free”; all they need to do is find it.

Conversely, participants were also critical of online health information. All participants explained that search results may not always be trustworthy or relevant to their individual circumstances. They believed it was possible to encounter misinformation dispersed amongst sources that seemed legitimate, making misinformation an inevitable part of searching.

Participants cautioned that the accessibility of health information could encourage self-diagnosis and hypochondria. Self-diagnosis was considered risky, especially given the amount of untrustworthy information and likelihood of misdiagnosis, and “if you get it wrong, there can be some pretty big consequences”. Furthermore, they identified the risk of catastrophising and obsessing over potential health issues because “people can jump to the worst conclusions…and start spiralling”. Participants raised the feedback loop created by the “endless scroll”, where they felt compelled to keep looking through search results because of the infinite amount of seemingly relevant information.

### Applying online health information

Participants reported reflecting on the health information they found online. If they felt satisfied that they “didn’t have anything serious”, they would not act further. They may follow simple lifestyle solutions suggested but would not act on the advice of online health information beyond this without consulting parents and/or health professionals.

If it recommends a doctor, I’d probably think about it first, like “Is it worth the money?”. If I see stuff like “eat this instead” or “drink water”, I would try that first, but then obviously if it doesn’t work, that’s when I see a doctor. (P6 - Female, aged 12)

#### Seeing a health professional

Participants may ask their parents about seeing a health professional after finding health information online if their symptoms were severe and/or the information indicated a serious disease, “like if it says cancer”. They may also seek health professional advice if they did not attain their desired outcome and felt results were confusing or inconclusive. They explained they would “assess if it was worthwhile” in terms of time and money to see a health professional for their pain, symptoms and search results. All participants explained this decision was made in conjunction with their parents, who ultimately assessed their need to see a health professional.

Participants rarely discussed online health information with health professionals, even if an appointment occurred after finding information online. They believed search results were not “useful” for the health professional’s diagnosis and therefore unnecessary to share as they would receive superior information during their appointment. Some refrained from disclosing health information searching to avoid embarrassment or judgement because they believed health professionals would dismiss online health information: “I remember the GP having a bit of a chuckle and being like, ‘Never trust what you read online’”. They also reported health professionals rarely asking if they had used online health information.

 Nevertheless, participants reported they were not against this disclosure and that it could improve their independence and self-efficacy to have such conversations with health professionals. They believed health professionals could provide guidance for future searching, as one 13-year-old male recommended, by providing “a list of verified online sources that are good for searching with the right information.”

#### Searching after seeing health professional

All participants favoured health professional advice over online health information. They placed trust in the qualifications and training of health professionals, reporting: “They’ve studied for a lot longer and helped more people than Google”. Participants appreciated that appointments provided individualised health advice that the internet could not replace.

Participants reported searching for online health information after health consultations to learn more or understand health professionals’ advice. When searching online post-appointment, participants sometimes found conflicting information. However, they always conceded that health professional advice was more relevant and trustworthy.

## Discussion

This study identified the ways adolescents access, appraise, understand and apply online health information. Adolescents displayed self-efficacy across these domains of digital health literacy and had many of the essential capabilities required to use this information effectively. We found adolescents access online health information both unintentionally on social media and intentionally using search engines. They appraise the medium, source and content of the information using strategies based on heuristics taught at school for general internet searching. Some of these strategies are unsuitable for the digital health information context, thus diminishing self-efficacy and capability. Adolescents sometimes encounter terminology that makes online health information hard to understand or appraise, but rarely act on information without parental or health professional advice.

**Fig. 1:**
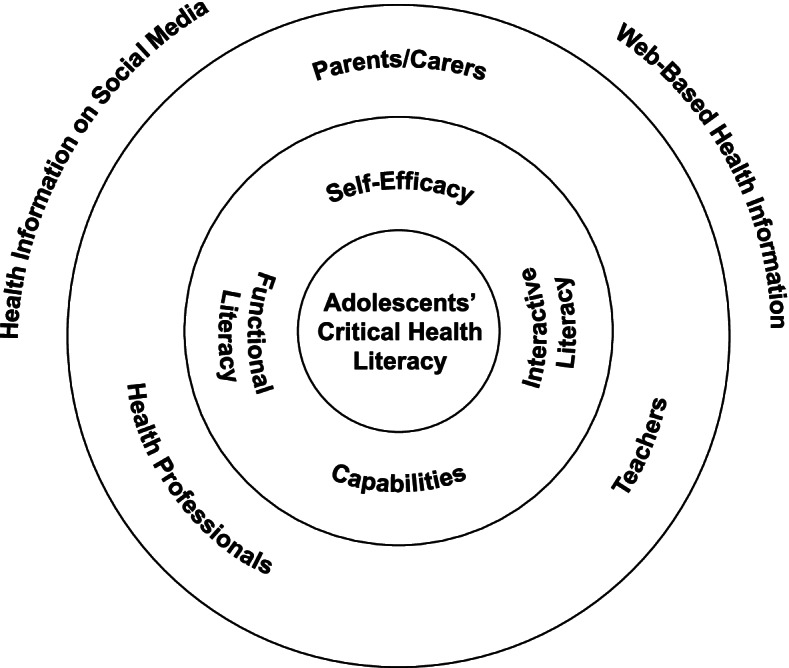
Influences on adolescents’ critical health literacy, capabilities and self-efficacy

The combination of an eHEALS survey, observed practical search task and follow-up interview, however, revealed the discrepancy between adolescents’ perceived and actual digital health literacy. Adolescents generally perceived their digital health literacy to be higher than they demonstrated in the search task, with many reporting using strategies to search and appraise health information but not demonstrating or understanding these strategies in practice, in line with previous studies [30, 37]. Additionally, all participants demonstrated similar search and appraisal behaviour no matter how competent they rated themselves in the eHEALS measure [30]. This suggests that adolescents cannot accurately identify their digital health literacy skills and whilst they may have a high sense of self-efficacy, it does not always reflect capability. We outline this conceptual relationship in Fig. 1.

When conceptualised using Nutbeam’s health literacy levels, our participants had functional and interactive literacy but lacked the critical health literacy required for optimum online health information use [18]. Accessing and understanding simple health information was not a functional challenge for adolescents during the search task and they reported rarely facing interactive health literacy challenges because they were selective about applying health information to personal concerns without approval from parents or health professionals [20]. Here, the adolescents’ self-efficacy was well founded, as awareness of their capacity ensures they are not making unsuitable health decisions based on information seen online. The key challenge pertains to critical health literacy required to consistently ascertain relevance and trustworthiness of online health information; the appraisal strategies adolescents use can be unreliable and generic.

Unreliable appraisal strategies that adolescents use include focusing on design and graphical elements of websites [38, 39], selecting sites with “health” in the results listing preview and assuming websites with .org domains include reliable and relevant information [23]. Adolescents also rely on the Google search engine to ascertain relevance and trustworthiness of information sources, selecting a website due to its ranking in the results. Here, adolescents are trusting the indirect source of information (i.e., Google) rather than appraising the information itself [23]. The use of these simple heuristics indicate adolescents are unaware of how website design and search engine algorithms are optimised to direct and keep digital traffic on certain webpages [37, 40, 41]. Familiarity (or lack thereof) also guides appraisal strategies as adolescents may base website selection on previously used sources [23, 37]. Social media specific strategies, such as considering the number of Likes on a post - the aggregated rating of the information across multiple, potentially unknown users - may also lead to inaccurate assessments of health information [39].

Adolescents practise these strategies indiscriminately, without critically analysing the complex factors that make online health information trustworthy. This was particularly the case with strategies appropriated from general internet heuristics rote-learnt at school for academic assignments. Adolescents would state that they knew .gov and .org domains were trustworthy because they were taught this at school, but were unable to explain what makes them more trustworthy than other domains. Similarly, adolescents knew to identify authors of information to ascertain trustworthiness, but did not know what different credentials signified nor how they qualified authors to provide trustworthy health information.

This automaticity in appraisal was also seen in reasoning about why social media was less trustworthy than other sources of online health information; on the basis the public can share unverified information. This appraisal strategy did not take into consideration that sharing of unverified health information occurs on other traditional online sources too, such as commercial websites that are not created by health professionals or organisations [7]. Nor does this strategy acknowledge that health information on social media can be as trustworthy as information found elsewhere on the internet, depending on the account and intentions behind the post (e.g., public health authorities using social media platforms for health promotion) [42, 43]. It is noteworthy that despite many of our participants reporting this belief, they also explained they would appraise the content of health information on social media in a similar way to web-based health information. A number of participants also preferred health information on social media, given it was easier to understand and provided information opportunistically.

Even though adolescents may not have fully developed critical health literacy, they practise strategies that reflect many elements of it [44]. They seek official and government websites, search for terms they do not understand, and cross-check and compare health information from multiple sources to ensure trustworthiness. [15, 45, 46]. They have measured attitudes towards online health information generally, aware of its benefits (including ease of use and accessibility [9]) and challenges and risks (the overwhelming abundance of information that may be difficult to understand, untrustworthy and irrelevant to their concerns [9]). They also display self-efficacy and awareness of the inherent issues with online health information by identifying the risks of self-diagnosis, hypochondria and health anxiety. Ultimately, adolescents have a desire to improve their digital health literacy as they appreciate that online health information provides them with the opportunities to learn more about their bodies and health states, and increase their self-efficacy [15].

Fostering critical health literacy in adolescents will ensure they have the ability to analyse and act on health information to make important health decisions [18, 47]. For this, adolescents need to be equipped with the ability to critically engage and appraise online health information on a case-by-case basis. Interventions need to support development of the most advanced cognitive and literacy skills so adolescents can learn to discriminate between varying sources of information, critically analyse meaning and relevance, and use information to exert greater control over a range of health determinants [18]. Interventions should focus on improving the self-efficacy of young people as it is pivotal in the adoption and execution of healthy behaviours and a predictor of digital health literacy [29].

Involving adolescents in the design and development of such interventions via co-design may further improve efficacy and uptake, whilst also empowering young participants and celebrating their knowledge and creativity. Schools, as identified by our participants and other studies, have opportunities to foster adolescents’ digital health literacy through the curriculum and supportive environment [48]. During healthcare, health professionals should assume adolescent patients use online health information and discuss it with them. Exploratory targeted interventions are already indicating it is possible to draw the attention of adolescents to critical aspects of internet searching and improve competence [30].

### Limitations

The simulated nature of the search task may not have allowed participants to accurately demonstrate their search and appraisal strategies as they were not experiencing the symptoms and related health anxiety. This may explain discrepancies between perceived and actual digital health literacy, and why the sophisticated strategies discussed in follow-up interviews were not demonstrated in search tasks. It is also difficult to disentangle the influence of social desirability bias on interview responses. For example, participants reported not acting on online health information but previous research found adolescents change health behaviours based on health information seen online [49].

Measurement of digital health literacy also poses a limitation in this study as the eHEALS, a self-report measure, may be influenced by other competencies, such as reading comprehension and self-efficacy [29, 30]. Whilst our search task observation checklist provided an assessment of digital health literacy, objective measures validated for adolescent assessment are required.

## Conclusions

This study has highlighted the need to improve adolescents’ critical digital health literacy. Whilst they already practise some important search and appraisal strategies when using online health information, higher level, critical health literacy skills will ensure they consistently use this information appropriately. Optimising digital health literacy in young people is particularly important in the context of their high internet usage and the recent rise of online health misinformation accompanying the COVID-19 pandemic. Strengthening these digital health literacy skills in adolescence will ensure they are carried through into adulthood, improving future health and wellbeing. Most importantly, adolescents want to improve their self-efficacy and digital health literacy so they can confidently appraise the online health information they encounter on social media or search for on the internet. Co-designed interventions and health provider involvement are required to build critical literacy capacity amongst adolescents and empower them to be active agents in their health.

## Supplementary Information

**Additional file 1.** Practical task and interview questions.

**Additional file 2.** Search/appraisal observation checklist.

## Data Availability

The datasets generated and/or analysed during the current study are not publicly available due to the sensitive age of the study participants (12-17 years). Parents have not given their informed consent and participants have not given their informed assent for their data to be shared. The Sydney Children’s Hospitals Network Human Research Ethics Committee has not provided approval for the sharing of this data. Contact corresponding author Associate Professor Karen Scott (karen.scott@sydney.edu.au) regarding data requests.
