# Supplementary material for: Adolescents’ self-efficacy and digital health literacy: a cross-sectional mixed methods study
Source: BMC Public Health. 2022 Jun 20;22:1223. doi: 10.1186/s12889-022-13599-7 (PMC9207829; doi:10.1186/s12889-022-13599-7)
Supplement: Supplementary file 1 — Additional file 1. Practical task and interview questions. [file 12889_2022_13599_MOESM1_ESM.docx]

Additional File 1

**Practical task and interview questions**

Thank you for agreeing to be a part of our study. We really appreciate you taking the time to share some of your experiences with us related to your use of online health information.

**Part 1: Concurrent think along protocol:**

The researcher asks the participant to look up online health information on specific symptoms, using the following script:

*“Imagine you have been experiencing tummy pains and you often feel bloated. You have also lost quite a bit of weight without trying. How would you look for information about this on the internet?”*

The participant searches for the desired online health information while verbally relaying their reasoning behind their decisions and movements between web pages. This verbal relaying is prompted by semi-structured questions form the interviewer to ascertain as much explanation as possible behind their search and appraisal of online health information. Examples of these semi-structured questions are outlined below.

***Search task semi structured questions:***

*“Can you please tell me why you:*

- *Used this search engine?*
- *Used these search terms?*
- *Selected this link?*
- *Didn’t select this link?*
- *Selected this piece of online health information?*
- *Rejected this piece of online health information?*
- *Looked at this section of the online health information?”*

*“Can you please tell me:*

- *How trustworthy you think this online health information is? What made you decide that?*
- *How relevant you think it is to the health topic you were looking up? What made you decide that?”*

**Part 2: Follow-Up Semi-Structured Interview Questions**

Following the search, the researcher asks semi-structured interview questions to ascertain the participant’s reasoning behind their appraisal process, and to determine what the participant believes may help with searching/appraisal in the future. The semi-structured questions are outlined below.

| **#** | **General Question** | **Probing Question(s)** |
| --- | --- | --- |
| **1** | Why were you interested in taking part in our study about online health information?  *Providing their opinion of online health information and addressing motive for study participation* |  |
| **2** | Think back over the past month to a time when you **used online health information:** can you tell me about your **experience**? | - **Why** did you use it? - **How** did you search? - **What** **sources** did you use? - **What** **devices** did you use? |
| **3** | How **typical** is this experience for you?  *Ascertaining if they look up health information in the same way when performing separate searches* | - Do you **use different ways** of searching for **different types** of health information? - **How often** do you look? |
| **4** | When you consider your experience using online health information, do you usually **find** the information you’re looking for?  *Understanding how they view their ability to find online health information* | - How do you know **when to stop**? - How do you **feel** when you’ve **found/ not found** information you were looking for? - What do you do when you can’t find information? - Has anyone **helped you**? - Have you **helped others**? - **What type of help do you need?** |
| **5** | When you consider your experience using online health information, do you usually **understand** the information you find?  *Ascertaining the good/bad aspects of resources accessed by adolescents* | - What do you think **helps you**? - What **stops you**? - How do you **feel** when you **understand/don’t understand** information? - Has anyone **helped you**? - Have you **helped others**? - **What type of help do you need?** |
| **6** | Thinking about online health information you’ve found, how do you decide whether it is **trustworthy** and **relevant**? | - Do you need help finding trustworthy information? - **What** has been your experience in finding **relevant** information **?** *(Lifestyle etc)* |
| **7** | Let’s consider the current situation with **COVID-19**: have you used online health information to **understand** it? Tell me a bit about that.  *Ascertaining if their search habits have been influenced by the pandemic* | - How did you **search**? - What did you find? - How **helpful** was it? - How did it make you **feel**? |
| **8** | What about **social media**: can you think of a time when you used social media to learn about your health?  *Behaviours/Environmental Influences* | - **What** **types** of social media did you use? *(Instagram, Facebook, Snapchat, Tiktok, YouTube, Twitter)* - **How** is online health information **presented**? - What **type of content** do you access? *(Influencers, Peers, Reputable Organisations)* - What is the **benefit/risk** of finding health information on social media? |
| **9** | What do you do **after** you find online health information?  *Behaviours/Environmental Influences* | - How do you **decide** what to do with it? - Do you **tell others** about it? *(Health Professionals, Parents/Carers, Friends)* - How do others’ **opinions** **influence** what you do with it? - Have you ever **shared** a post on social media about online health information? |
| **10** | Let’s say you’ve looked at online health information: how do you **decide** whether you need to **see a health professional**? *(Doctor, dentist, physio, psychologist)*  *Behavioural Influences – What enables/hinders actions* | - Do you look **before** you see a health professional? - Do you look **after** you see a health professional? - Have you ever **changed your treatment** after reading online health information? - What is the **benefit/risk** of using online health information? |
| **11** | Thinking back to when you **saw a health professional**: did you **tell** them about using **online health information**? Why? | Has your health professional **asked** you about your use of online health information?   - What did/would you say? - How could health professionals **help** you in use online health information? *(Provide advice? Reliable resources?)* |
| **12** | Thank you for reflecting on your experiences! Is there anything else you’d like to add? |  |
