# Supplementary material for: Adolescents’ self-efficacy and digital health literacy: a cross-sectional mixed methods study
Source: BMC Public Health. 2022 Jun 20;22:1223. doi: 10.1186/s12889-022-13599-7 (PMC9207829; doi:10.1186/s12889-022-13599-7)
Supplement: Supplementary file 2 — Additional file 2. Search/appraisal observation checklist. [file 12889_2022_13599_MOESM2_ESM.docx]

Additional File 2

**Search/appraisal observation checklist**

| **Task** | **Digital skills** |
| --- | --- |
| Participant ID |  |
| Search engine used | Google  Bing  Yahoo  Other (please specify) |
| Search terms entered (describe in depth) |  |
| Search terms entered (select all that apply) | Scenario 1:  Tummy/stomach pains  Bloating  Losing/lost weight/underweight |
| Position of 1^st^ chosen link in search results (describe in depth) |  |
| Position of 1^st^ chosen link in search results (select one) | Search engine pop-up/suggestion  Advertisement  First search result on first page (after advertisements (ads))  Second search result on first page (after ads)  Third search result on first page (after ads)  Fourth search result on first page (after ads)  Last search result on first page  On second page  Other |
| Decision about trustworthiness and relevance of 1^st^ chosen link |  |
| Parts of website spent time on |  |
| Number of clicks within website to achieve decision |  |
| Length of time within website to achieve decision |  |
| If necessary, repeat above | |
| Total time spent on search |  |
| Total number of webpages visited |  |
| Participant digital literacy level  *Assign a point for each of the following demonstrated in the search task:*   1. *Used search terms relevant to scenario* 2. *Used government and official health websites* 3. *Investigated author of source* 4. *Investigated bias/funding/conflict of interest of source/hidden agenda of source* 5. *Investigated date of source* 6. *Investigated privacy policy* 7. *Cross checked information* 8. *Investigated meaning of confusing words further* 9. *Looked for HoN seal* 10. *Spent an acceptable amount of time on the search (between 2-15 minutes)*   *Dock points if the following demonstrated:*   1. *Relying solely on 1 source of information* 2. *Relying solely on Google Suggestions/Snippets* 3. *Using untrustworthy websites, websites with ads, non-official/government websites, unknown websites* 4. *Relying solely on non-health professional authors* 5. *Looking for specific/too-good-to-be-true treatments* | Low = 4 or less points  Moderate = 5-7 points  High = 8+ points |
